# An analysis of the left top pulmonary vein and comparison with the right top pulmonary vein for lung resection by three-dimensional CT angiography and thin-section images

**DOI:** 10.1007/s11604-023-01424-z

**Published:** 2023-04-11

**Authors:** Makiko Murota, Takashi Norikane, Yuka Yamamoto, Mariko Ishimura, Katsuya Mitamura, Yasukage Takami, Kengo Fujimoto, Katashi Satoh, Naoya Yokota, Yoshihiro Nishiyama

**Affiliations:** 1 Department of Radiology, Faculty of Medicine, Kagawa University, 1750-1 Ikenobe, Miki-Cho, Kita-Gun, Kagawa 761-0793 Japan; 2 Department of Radiology, Diagnostic Imaging Center, Utazu Hospital, Utazu-Cho, Ayauta-Gun, Kagawa Japan; 3 Department of General Thoracic Surgery, Faculty of Medicine, Kagawa University, Kita-Gun, Kagawa Japan

**Keywords:** Pulmonary veins, Three-dimensional CT pulmonary angiography, Video-assisted thoracic surgery, Anatomy, Lung cancer

## Abstract

**Purpose:**

The right top pulmonary vein (RTPV) is defined as an anomalous branch of the right superior PV (SPV) draining into the PV or left atrium (LA). Several previous reports have described the RTPV, but only a few have mentioned the left top PV (LTPV). The present study aimed to evaluate the branching patterns of the RTPV and LTPV using thin-section CT images and three-dimensional CT angiography (3D-CTA).

**Materials and methods:**

This study included 1437 consecutive patients for evaluation of the right side and 1454 consecutive patients for the left side who were suspected of lung cancer and underwent CTA. We assessed the presence of each RTPV and LTPV and their branching patterns on the CTA images. When the RTPV or LTPV was identified, the maximum short-axis diameter was measured.

**Results:**

RTPV was found in 9.1% (131/1437), whereas LTPV was found in 2.9% (42/1454) of the patients. RTPV was also observed in 17.1% (7/41) of LTPV cases, except for one case in which the right side could not be evaluated. The most common RTPV inflow site was the right inferior PV (IPV) in 64.9% (85/131) of the patients, whereas that of the LTPV was the left IPV in 100.0% (42/42) of the patients. The mean diameter of the RTPV and LTPV was 3.3 mm (range, 1.3–7.5 mm) and 2.4 mm (range, 0.9–6.3 mm), respectively (P < 0.01).

**Conclusion:**

The top PV branching pattern variations can be evaluated using thin-section CT and 3D-CTA images. RTPV is not a rare finding, and LTPV should also be identified in lung cancer cases scheduled for resection.

## Introduction

Understanding the pulmonary vein (PV) branches and their variations is the key to successful anatomical lung resection for lung cancer. Advanced imaging techniques, such as three-dimensional CT angiography (3D-CTA), have enabled surgeons to easily and precisely confirm preoperative anatomical information for the safety of surgical procedures, including video-assisted thoracic surgery (VATS) lobectomy and limited resection [1–3]. Several investigators have reported variations in PV branching patterns using CT images and the 3D-CTA [4–7]. One particular pulmonary venous variant is the right top pulmonary vein (RTPV), which passes through the dorsal aspect of the bronchus intermedius and drains caudally from the veins of the right upper lobe. Through reports of preoperative catheter ablation and lung cancer using CT and MRI, the RTPV has become known and is being identified more [8–14].

On the left side, a variation similar to the RTPV [15–17], referred to as the left top pulmonary vein (LTPV), has been described in a few case reports but has not been studied in detail. Therefore, this study aimed to evaluate the branching patterns of the RTPV and LTPV using thin-section CT images and 3D-CTA.

## Materials and methods

### Patients

**Fig. 1:**
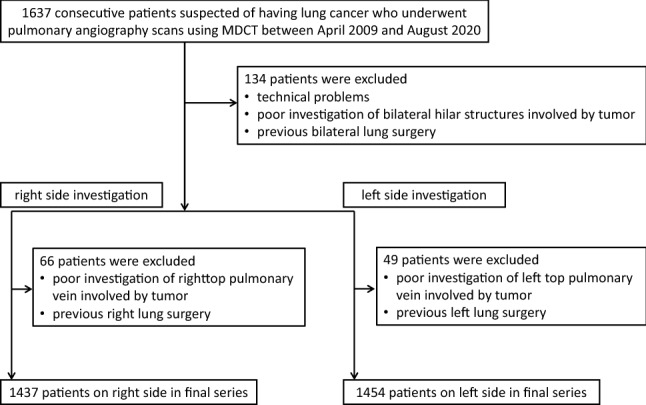
Study flowchart

The ethics committee of our hospital approved this retrospective study and waived the need for obtaining consent from individual patients. A total of 1637 consecutive patients suspected of having lung cancer who underwent pulmonary angiography using multidetector row CT (MDCT) between April 2009 and August 2020 were retrospectively reviewed. Of these, 134 patients were excluded from this study because of technical problems, poor investigation of bilateral hilar structures involved by tumor, and previous bilateral lung surgery. A total of 66 patients were excluded from the right lung analysis because of poor investigation of the right hilar structures involved by tumor and previous right lung surgery, and 49 patients were excluded from the left side analysis because of poor investigation of the left hilar structures involved by tumor and previous left lung surgery. The final study population included 1437 patients (951 men, 486 women; mean age, 68.6 years; age range, 13–92 years) for evaluation of the right side and 1454 patients (964 men, 490 women; mean age, 68.7 years; age range, 13–92 years) for the left side (Fig. 1). Of the 1437 patients who underwent right side evaluation, 741 underwent pulmonary resection (upper lobectomy in 279 patients, middle lobectomy in 72, lower lobectomy in 134, upper and middle lobectomy in 3, middle and lower lobectomy in 13, upper lobe segmentectomy in 36, middle lobe segmentectomy in 1, lower segmentectomy in 59, upper lobe segmentectomy and lower lobe segmentectomy in 4, wedge resection in 132, pneumonectomy in 1, and others in 7), whereas 696 were inoperable cases or underwent follow-up. Of the 1454 patients who underwent left side evaluation, 506 underwent pulmonary resection (upper lobectomy in 183 patients, lower lobectomy in 112, upper lobe segmentectomy in 76, lower segmentectomy in 28, upper lobe segmentectomy and lower lobe segmentectomy in 3, wedge resection in 90, pneumonectomy in 2, and others in 12), whereas 948 were inoperable or underwent follow-up.

### Contrast-enhanced MDCT

We used 64-detector MDCT (Aquilion 64, Canon Medical Systems, Tokyo, Japan), 128-detector MDCT (Brilliance iCT, Philips Healthcare, Cleveland, OH, USA), and 256-detector MDCT (Revolution CT, GE Healthcare, Milwaukee, WI, USA) scanners for imaging. The technical parameters used for the 64-detector MDCT were as follows: a detector row configuration of 0.5 mm, pitch of 53 (detector pitch 0.83), reconstruction increment of 0.4 mm, and section thickness of 0.5 mm. The corresponding values for the 128-detector were 0.625 mm, 106 (detector pitch 0.83), 0.5 mm, and 0.67 mm, and for the 256-detector MDCT were 0.625 mm, 254 (detector pitch 0.992), 0.625 mm, and 0.625 mm, respectively. An X-ray tube voltage of 120 kVp was used for the 64-slice and 128-detector MDCTs, and 60 keV was used for the 256-detector MDCT. We used an automatic exposure control for tube current in all examinations.

A dual-head power injector (Dual Shot GX, Nemoto Kyorindo, Tokyo, Japan) was used for all patients to administer the contrast material iohexol (Omnipaque 350, GE Healthcare Pharma, Tokyo, Japan) or iopamidol (Iopamiron 300, Bayer Yakuhin, Osaka, Japan) via the cubital vein. In patients weighing at least 55 kg, 100 mL of contrast medium (CM) iohexol (350 mg iodine/ml) was injected at a rate of 3.3 ml/s, and scanning was performed 18 s after the start of CM injection. In patients with body weight ranging from 44 to 55 kg, 85 ml of CM iohexol (350 mg iodine/ml) was injected at a rate of 3.3 ml/s, and scanning was performed 15 s after the start of CM injection. In patients weighing less than 44 kg, 85 ml of CM iopamidol (300 mg iodine/ml) was injected at a rate of 3.3 ml/s, and scanning was performed 15 s after the start of CM injection. For 256-detector CT, patient-weight-dependent CM at a dose rate of 24 mgI/kg/s (iopamidol: Iopamiron 300 mg iodine/ml or iohexol: 350 mg iodine/ml) was used with a 15 s injection time, and scanning was performed 20 s after the start of injection. A mixed saline chaser (CM 30%, saline 70%; injection time 15 s) and a normal saline chaser (injection time 5 s) at the same injection rate were administered. Another protocol for pulmonary artery (PA) and PV separation images determined from the time-density curve using a test bolus dose was applied. The injection rate was 4 ml/s, with a 20 ml test bolus injected prior to the main injection. The test injection determined the appropriate timing of the PA/PV scan. The PA/PV scan was performed after the start of the CM iohexol (350 mg iodine/ml) injection with 50 ml. The saline chaser was 40 ml and the injection rate was 4 ml/s. These three protocols were comparable for investigating the PA branching pattern in detail. The volume data obtained from the arterial phase were transferred to a workstation (Zio STATION, Ziosoft, Tokyo, Japan), where they were converted to a 3D-CTA format using the volume-rendering technique.

### Anatomy

**Fig. 2:**
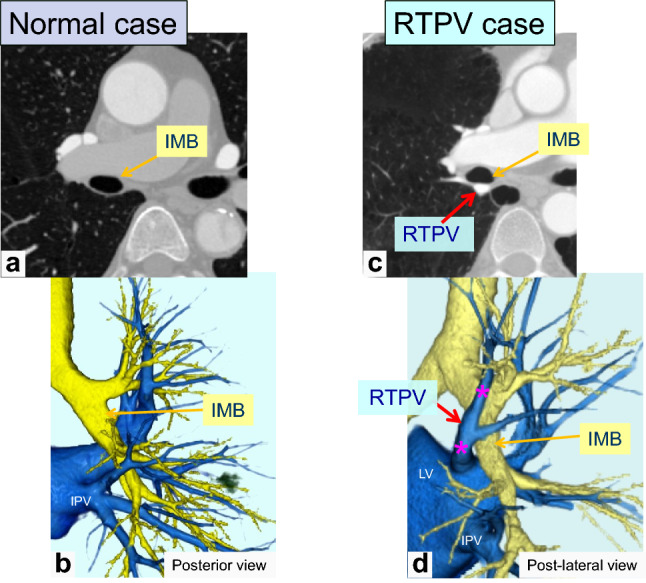
Thin-section and 3D-CTA images of a normal case and a case of right top pulmonary vein (RTPV). Thin-section CT (**a**) and 3D-CTA (**b**) images showing the normal pattern of the pulmonary vein. Thin-section CT (**c**) and 3D-CTA (**d**) image showing the RTPV

**Fig. 3:**
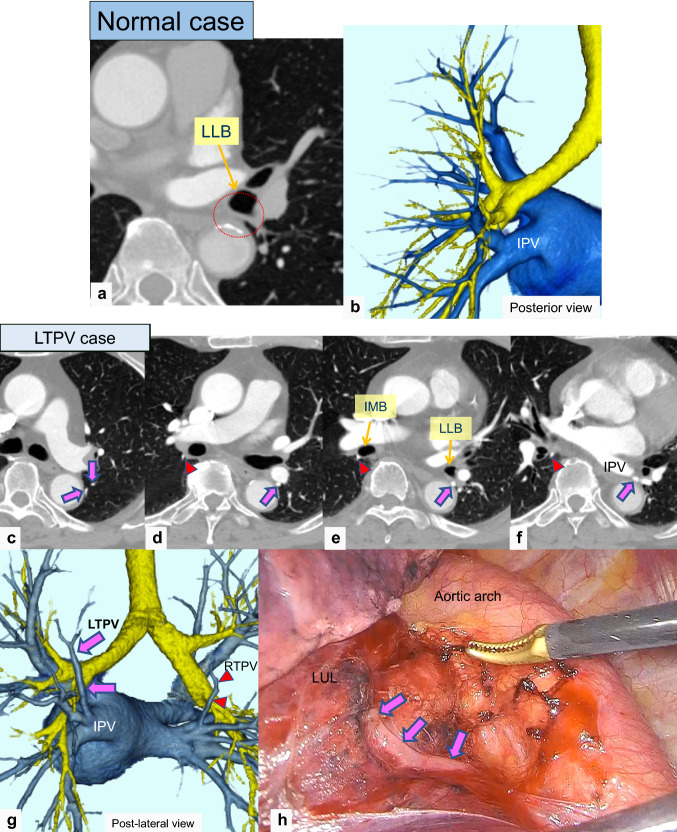
Thin-section and 3D-CTA images of a normal case and a case of left top pulmonary vein (LTPV). Thin-section CT (**a**) and 3D-CTA (**b**) images showing the normal pattern of the pulmonary vein. Thin-section CT (**c**–**f**) and 3D-CTA (**g**) images showing the LTPV (pink arrow) with the RTPV (red arrowhead). The intraoperative image (**h**) of a 70’s-year-old man with lung cancer of the left upper lobe showing the LTPV

The RTPV is defined as the anomalous branch of the right upper lobe vein, which passes through the posterior surface of the bronchus intermedius on axial CT images and drains into the PV or left atrium (LA) [12, 13] (Fig. 2). Regarding the LTPV, we defined it as an anomalous branch of the left apicoposterior segmental vein that passes mainly through the posterior surface of the left lower bronchus and drains into the PV or LA, similar to the RTPV (Fig. 3).

### Image analysis

Thin-section images were reviewed at a width of 1600 HU and level -200 HU window settings with paging on a viewer (EV insite, PSP Corporation, Tokyo, Japan). The 3D-CTA images were interpreted by rotating images on the same viewer. The window, level, and opacity of the volumes were subjectively selected to optimize the visualization of the PV. In the present study, the RTPV and LTPV and their branching patterns were identified meticulously using 3D-CTA and thin-section images of the same viewer. Two board-certified thoracic radiologists with 14 and 24 years of experience independently reviewed each CT image. In case of discrepancy over branching patterns between the investigators, the case was again reviewed with 3D-CTA and thin-section images, and a consensus was reached to avoid interobserver variability. When the RTPV or LTPV was identified, the maximum short-axis diameter was measured at the level posterior to the bronchus intermedius [10] or the left lower bronchus.

### Statistical analysis

Statistical analyses were performed using SPSS statistics software (Version 28, 2021; IBM Corp. Armonk, NY). The diameter data on the CT images were compared using the Mann–Whitney U test. Statistical significance was set at P ≤ 0.05. Interobserver agreement regarding the presence or absence of CT findings was analyzed by calculating the kappa statistic on the assessments made prior to agreement by consensus by a thoracic radiologist who reviewed the images. Interobserver agreement was classified as follows: excellent (κ = 0.81–1.00), substantial (κ = 0.61–0.80), moderate (κ = 0.41–0.60), fair (κ = 0.21–0.40), and poor (κ = 0–0.20).

## Results

RTPV was found in 9.1% (131/1437) of the patients, whereas LTPV was found in 2.9% (42/1454) (Figs. 2 and 3). The interobserver agreement for identifying the presence of RTPV and LTPV was excellent and moderate (κ = 0.86 and κ = 0.43, respectively). RTPV was also observed in 17.1% (7/41) of LTPV cases (Fig. 3), except for one case in which the right side could not be evaluated.

**Fig. 4:**
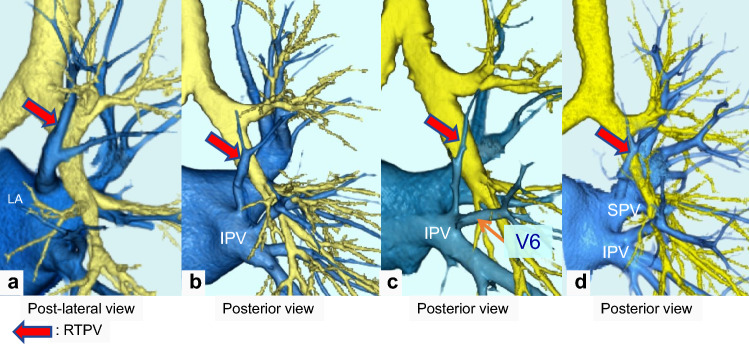
Branching pattern types of the right top pulmonary vein (RTPV: red arrow). **a** shows RTPV draining directly into the left atrium (LA); **b** shows RTPV draining into the right inferior pulmonary vein (IPV); **c** shows RTPV draining into the right IPV with V6; and **d** shows RTPV draining into the right superior pulmonary vein (SPV)

**Fig. 5:**
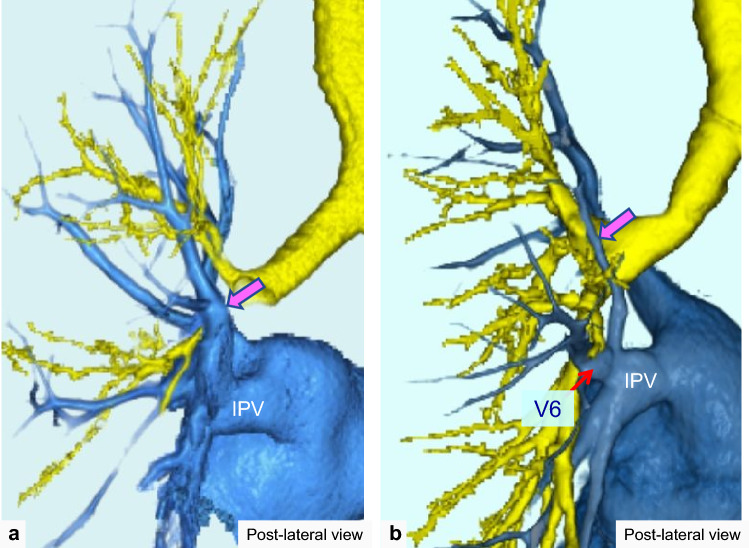
Branching pattern types of the left top pulmonary vein (LTPV: pink arrow). **a** shows LTPV draining into the right inferior pulmonary vein (IPV) and **b** shows LTPV draining into the right IPV with V6

The inflow site of the top PVs on CT images is shown in Table 1, and each inflow site is also shown with regard to the RTPV and LTPV with or without the V6 branch. Figures 4 and 5 show the RTPV and LTPV branching patterns according to the 3D-CTA and thin-section CT images. The most common inflow site of the RTPV was the right inferior pulmonary vein (IPV) in 64.9% (85/131) of the patients, with branches of V6 in 50.4% (66/131), and the second most common was the LA in 22.1% (29/131) of the patients. In contrast, the inflow site of LTPV was the left IPV in 100.0% (42/42) of the cases, which was similar to that of V6 in 59.5% (25/42) of the cases. As for “other”, there was one case of RTPV with V6 draining into the right middle lobe vein and the right middle lobe vein draining into the LA alone, separately from the superior pulmonary vein.

**Table 1:** The inflow site of top pulmonary veins

	Number of patients (%)		κ
Right side	n = 1437		
Right top pulmonary vein	131 (9.1)		0.88
Inflow site			
IPV	85 (64.9)		
	IPV (without branches of V6)	19 (14.5)	
	IPV (with branches of V6)	66 (50.4)	
LA	29 (22.1)		
	LA (without branches of V6)	16 (12.2)	
	LA (with branches of V6)	13 (9.9)	
SPV	16 (12.2)		
	SPV (without branches of V6)	9 (6.9)	
	SPV (with branches of V6)	7 (5.3)	
Other	1 (0.8)		
Left side	n = 1454		
Left top pulmonary vein	42 (2.9)		0.43
Inflow site			
IPV	42 (100.0)		
	IPV (without branches of the V6)	14 (33.3)	
	IPV (with branches of the V6)	25 (59.5)	
	Other courses to IPV	3 (7.2)	

*IPV* inferior pulmonary vein, *LA* left atrium, *SPV* superior pulmonary vein

**Fig. 6:**
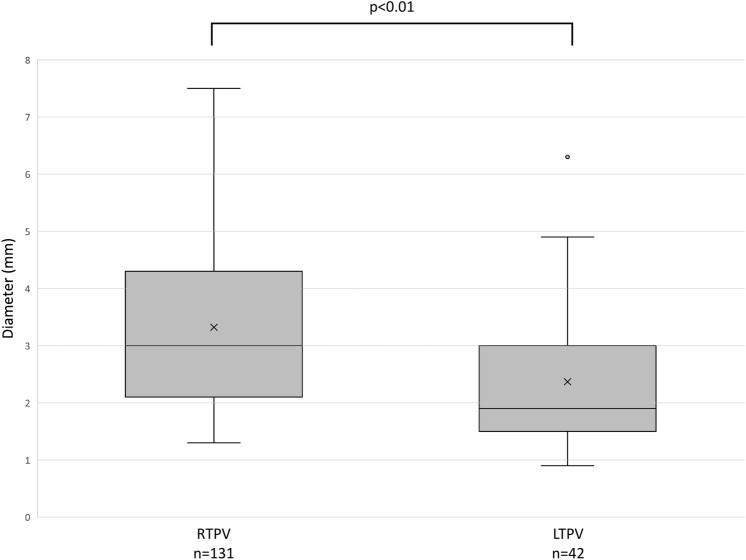
Diameter of the right top pulmonary vein (RTPV) and left top pulmonary vein (LTPV). The diameter of the LTPV was significantly smaller than that of the RTPV. The Mann–Whitney U test was used for analysis

The mean diameter of the RTPV and LTPV was 3.3 mm (range, 1.3–7.5 mm) and 2.4 mm (range, 0.9–6.3 mm), respectively (P < 0.01) (Fig. 6).

## Discussion

To the best of our knowledge, this is the first detailed study on LTPV using 3D-CTA and thin-section CT images in a large number of subjects. The present study showed the incidence and radiological features of LTPV compared to RTPV. The incidence of LTPV was 2.9% on CT images, whereas RTPV was 9.1%. The frequency of RTPV occurrence on CT images has been reported to be 3.3–9.1% [8, 10, 13, 14]. The frequency of RTPV tends to be low when CT axial images are analyzed with thicker section (5–10 mm) [8, 10, 14], while it tends to be high when CT images are analyzed with thinner sections [13], as in the present study. The difference in frequency between studies may be because of the difference in CT slice thickness used [10]. In their study, Aslan et al. detected RTPV in 2.2% of the study population; however, their investigation of RTPV was described only by its drainage into the LA, not including the right superior PV or IPV [11]. There are only a few case reports on the LTPV [15–18]. In this study, RTPV was observed in 17.1% of the LTPV cases, although the reason is unclear.

The drainage patterns of the RTPV were observed as draining into the right IPV, LA, and superior pulmonary vein (SPV), as described in previous reports. However, the frequency of RTPV drainage patterns differs among reports. Regarding the inflow site of the RTPV, previous investigations using 3D-CTA and thin-section CT [13] suggested that the SPV tends to be less common (9.7–12.2%) and the LA more common (22.1–35.0%), whereas those using only axial 5–10 mm images or non-enhanced CT [10, 14] reported that the LA tends to be less common (1.9–4.9%) and the SPV more common (53.7–55.8%). These results are similar to our findings. 3D-CTA images can more clearly depict the pulmonary vessels [6, 7, 11–13, 19]. It is difficult to identify the exact course of the RTPV using thicker images alone. The present study showed that all LTPVs flowed into the left IPV. The lingular vein, a vein of the left upper lobe, occasionally drains into the left IPV [6, 7]. Regarding the LTPV, a few case reports have been published, and in all of these cases, as in our study, the LTPV drained into the left IPV [15–18].

Anatomical variations of the PV, such as the RTPV or LTPV, can cause unexpected intraoperative bleeding. Furthermore, venous ligation of a nonresected segment or lobe can cause postoperative complications and residual lung dysfunction since dissection of the RTPV or LTPV can cause infarction or necrosis of the residual lung parenchyma [15]. For safe pulmonary resection of the upper and lower lobes, it is important to determine whether the RTPV has branches of V6 because the RTPV is a branch of the PV of the upper lobe [10, 14]. The risk of vascular injury in patients with RTPV with branches of V6 during pulmonary resection has been described [10]. Thin-section CT images can provide detailed information regarding pulmonary vessels [19]. Therefore, in the present study, we confirmed not only the inflow site of the RTPV and LTPV but also whether they were present with branches of V6 on thin-section CT. For precise identification of the anatomical structures, not only 3D-CTA but also thin-section CT images contribute to a better evaluation of the branching pattern of PVs. Lickfett et al. discussed the RTPV using magnetic resonance angiography for catheter ablation [9]. Because of the nature of magnetic resonance angiography, it is not possible to correctly identify the origin of the PV and the presence or absence of the V6 branch [11].

In the present study, the mean diameter of the RTPV and LTPV was 3.3 mm (range, 1.3–7.5 mm) and 2.4 mm (range, 0.9–6.3 mm), respectively. There was a significant difference between the diameters of the LTPV and RTPV. The worse interobserver agreement for the LTPV than for the RTPV may be because the LTPV is thinner than the RTPV. Moreover, this might also be because the presence of the LTPV is less known or poorly recognized than that of the RTPV. A thick RTPV or LTPV may be the main drainage vein of the right or left upper lobe, and separation of such a PV could lead to congestion of the lung parenchyma [11, 13]. Miyamoto et al. considered RTPV thicker than 5 mm in diameter to be the main drainage vein of S2 [13]. Although the LTPV was thinner than the RTPV in this study, LTPVs thicker than 5 mm have also been observed. Therefore, LTPVs should be identified during investigations.

The present study has several limitations. First, it was a retrospective study that introduced inherent selection bias. Second, 64-detector MDCT, 128-detector MDCT, and 256-detector MDCT scanners were used for imaging. We analyzed the RTPV and LTPV using 3D-CTA and thin-section CT images using three CTA protocols. However, we consider any differences among them not to have affected the results because CT images are sufficient to investigate the PV branching pattern. Finally, we exclusively used 3D-CTA and thin-section CT images. It is possible that these may vary somewhat from the actual anatomical findings. However, a previous study has shown a good correlation between CT findings and confirmed intraoperative findings of PV branching pattern abnormalities [7].

In conclusion, in the present study, 3D-CTA and thin-section images provided precise preoperative information regarding the existence and branching patterns of RTPV and LTPV. This study is the first to describe the LTPV in detail. The incidence of LTPV was 2.9% on CT images, all LTPVs flowed into the left IPV, and the diameter of the LTPV was significantly thinner than that of the RTPV. Preoperative detailed identification of pulmonary vascular variations, such as RTPV and LTPV, by 3D-CT and thin-section CT, plays an important role in preventing avoidable complications. RTPV occurrence is not rare, and the presence of LTPV should also be investigated in lung cancer cases scheduled for resection.
